# Post-COVID-19 acute invasive fungal rhinosinusitis: a systematic radiological approach in the light of clinico-surgical characteristics

**DOI:** 10.1186/s43055-022-00880-3

**Published:** 2022-09-14

**Authors:** Ekhlas Shaban, Rasha Aly Saleh, Mostafa Ibrahim Ammar, Kamal Ebeid

**Affiliations:** 1 Department of Radiodiagnosis and Medical Imaging, Faculty of Medicine, Tanta University, Tanta, Egypt; 2 Otorhinolaryngology Department, Faculty of Medicine, Tanta University, Tanta, Egypt

**Keywords:** COVID-19, Rhinosinusitis, Invasive fungal sinusitis, CT, MRI

## Abstract

**Background:**

The objective is to describe the radiological imaging findings of post-COVID-19 acute invasive fungal rhinosinusitis (AIFRS), being familiar with the wide variety of imaging spectrum, thus raising the suspicion for early diagnosis. Methods: In this retrospective study, we reviewed the imaging findings in 54 patients with proven post-COVID-19 AIFRS who underwent endoscopic/surgical debridement from April 2020 to September 2021. Most of these patients presented with facial or orbital swelling or facial pain. Medical records with a special emphasis on radiological imaging (50 NCCT of the paranasal sinuses and 17 MRIs of the orbit) were reviewed regarding the degree of mucosal disease of sinuses, nasal cavity, and nasopharynx, extra sinus soft tissue infiltration, especially orbital and cerebral extension (parenchymal, cavernous sinus, vascular or neuro-invasion).

**Results:**

We reported findings in 54 patients with post-COVID-19 AIFRS, of whom 30 were men and 24 were women with a mean age of 48.06. Unexpectedly, infiltration of pterygopalatine fossa was found to precede mucosal opacification of sinuses nasal cavity and affection of nasopharynx. Out of 54 patients, 49 showed inflammatory changes involving pterygopalatine fossa, 29.6% of patients showed infiltration of orbital tissues, 22 patients suffered from a fungal invasion of the cavernous sinus and 3 patients had carotid artery involvement.

**Conclusions:**

Imaging findings of AIFRS significantly vary from subtle mucosal thickening of paranasal sinuses, up to orbital and intracranial extension with vascular thrombosis and neuroinvasion. The hallmark inflammatory tissue infiltration into the pterygopalatine fossa and facial soft tissue may precede mucosal disease.

## Background

Since breaking at the end of 2019, the COVID-19 pandemic continues to be a challenge on a global scale. While efforts are being maintained to understand the pathophysiology and treatment of COVID-19 aiming to contain the disease, many other health problems were brought to the surface on top of COVID-19. Some of these disorders may affect patients during active COVID-19 infection in the recovery period was variable sequelae. One of the most devastating diseases that were brought back to action in the COVID-19 era is acute invasive fungal rhino-sinusitis (AIFRS) [1].

The coincidence of diabetes mellitus, unintended consequences of high-dose steroid administration, possible immune effects of COVID-19 virus and possibly the use of contaminated industrial oxygen may all have a predisposing contribution to this new organism [2].

In AIFRS, the middle turbinate has always been thought to be the epicentre of infection in most cases from where it tends to spread to the rest of the nasal cavity and sinuses. Mucor has angioinvasive properties that enable it to rapidly progress through tissue borders beyond the limits respected in the immunocompetent status. Spreading along neurovascular bundles appears to be the highway for this pathogen, resulting in extensive tissue necrosis with consequent high mortality of 50–80% and high morbidity in those who survive [3].

The fundamental to successful management is early and aggressive medical and surgical treatment. Systemic antifungals should be administered and correction of underlying comorbidities. Surgical debridement is used for removing all necrotic tissue [4].

A biopsy is essential to confirm the diagnosis. However, the microbiological diagnostic pathway for AIFRS is special, since tissue grinding can lead to negative results because of the destruction of the hyphae, causing unsuccessful culture growth; therefore, it is important to inform the laboratory early about the suspicion of AIFRS to adapt the diagnostic technique. Recent developments tend to favour molecular diagnostic assays in order to accelerate the diagnosis [5].

The use of radiological investigations is indispensable to the initial diagnosis and guidance of management. Computed tomography (CT) is the workhorse for imaging the paranasal sinuses. MRI is used to evaluate the orbit, perineural spread along the trigeminal nerve, cavernous sinus involvement and cerebral extension [1]. We need more details on the common radiological findings of AIFRS described before. Imaging findings are variable. Sever sinonasal mucosal thickening with signs of inflammatory involvement just outside the paranasal sinuses and features of potential complications raise suspicion of the diagnosis. Mild sinonasal mucosal thickening is not specific to the diagnosis of the disease [6].

A systematic reporting approach with a structured checklist is helpful to not miss critical findings in the radiological report [1, 7].

The aim of this study is to describe radiological imaging findings of AIFRS in COVID-19 patients, being familiar with different radiologic characteristics, and raise the suspicion for early diagnosis.

## Methods

### Patients

This retrospective study was conducted in our University Hospital in the period between April 2020 and November 2021. Medical records of 54 confirmed COVID-19 patients with pathologically proved acute invasive fungal sinusitis were reviewed with a special emphasis on radiographic imaging. Demographic data and medical history of other comorbidities complete clinical examination including nasal endoscopy, and laboratory and histopathological findings were collected.

### Ethics approval

This study was approved by the local ethics committee.

### Method

Of 54 patients, 50 patients underwent NCCT examinations of paranasal sinuses (PNS); 37 patients (68.5%) underwent CT only, 13 patients (24.1%) underwent both CT and MRI, and 4 patients (7.4%) underwent MRI only.

#### Image acquisition

(a) Non-contrast computed tomography (NCCT) paranasal sinus protocol performed using a multislice CT Aquilion One, Toshiba Medical Systems, Otawara, Japan: The scan was obtained in the axial plane from above the frontal sinuses through the hard palate; slice thickness of 1 mm with no spacing, and field of view (FOV) 170–190 mm.

#### Image reconstruction

Reconstructions were performed using a bone algorithm (section thickness of 0.5 mm with 0.5-mm spacing). Multiplanar reformations were also completed in the coronal and sagittal plane using post-processing: The reconstructed images were transferred to the workstation (Vitrea Fx, Vital Images, USA). (b) MR imaging of the orbit was performed using a 1.5 T MR scanner (Signa16 channel, Excite, GE Healthcare, Milwaukee, WI, USA).

Orbit protocol was done for 17 patients: T1WI (TR/TE/ 400–644 ms/8–20 ms/), T2WI fat-saturated (TR/TE/, 3200–5000 ms/85–129 ms). Slice thickness/gap/FOV = 2–3 mm/0.5 mm/18 cm), diffusion-weighted imaging (DWI) and ADC with the following parameters *B* = 0, *B* = 1000 s/mm^2^ TR/TE = 10,000 ms/76.8 ms, slice thickness, 0.3 mm slice gap. Postcontrast T1WIs fat-saturated (except 2 patients with impaired renal function) were obtained 20–30 s after intravenous administration of 0.1 mmol/kg gadopentetate dimeglumine (Magnevist; Schering, Berlin, Germany).

Brain sequence: T2-weighted images parameters (TR: 6672 ms, TE: 147 ms); FLAIR (TR: 7432, TE: 118.6, inversion time (TI): 2200); field of view (FOV) 22 × 18 mm; matrix, 310 × 620; slice thickness, 5 mm; and slice gap, 0.4 mm.

#### Image analysis

Two radiologists and an otolaryngologist conjointly interpreted the studies (E.S.: 14 years of experience, R.S.: 15 years of experience, and K.E.: 14 years of experience in endoscopic surgery). The radiologists were blinded to patient clinical information and histopathology results. The readers graded the amount of mucosal disease in each of the sinuses (maxillary, frontal, sphenoid, ethmoid air cells), nasal cavity (anterior and posterior), and nasopharynx. The mucosal disease was scaled on a 5-point score (0 = subtle mucosal thickening, 1 < 25% opacified, 2 = 25%: < 50% opacified, 3 = 50%: < 75% opacified, 4 = 75%:100% opacified, 5 = mucocele [100% opacified with expansion]). The readers also documented the presence of extrasinus extension of the disease to the pterygopalatine fossa, periantral fat (anterior and posterior periantral), nasolacrimal duct, orbital fat (medial and inferior) and submucosa/bone of the hard palate. The presence of nasal septal mucosal ulceration was reported as either “present” or “not present”. The presence of intracranial extension (subdural, epidural, or brain parenchymal) with cavernous sinus involvement, abscess formation, bone dehiscence, arterial thrombosis, and/or venous thrombosis was also recorded.

### Statistical analysis

Analysis of the data was performed; number, mean ± standard deviation (SD), and frequency (percent) for categorical data were calculated. Chi-square test (with exact p value) is used to describe categorical data distribution. *P* < 0.05 was considered a significant level. The correlation of findings was tested using Pearson test. All statistical analyses were analysed using IBM SPSS software package version 25.0 (Armonk, NY: I BM Corp).

## Results

The demographic and clinical variables of the 54 patients included in the study were analysed. The age ranged from 12 to 73 years with the mean age of 48.06 ± 16.5 years, being 55.6% male and 44.4% female (Table 1). As expected, the most common predisposing conditions were diabetes mellitus. The second common comorbidity is hypertension in 27 patients (Table 2).

**Table 1:** Demographic data and clinical presentation of patients enrolled in the study

	Variable	Number	Percent
Age (12:73 Years)	Mean	48.06	
	Std. deviation	16.5	
Gender	Male	30	55.6
	Female	24	44.4
Clinical presentation*	Facial pain or loss of sensation	50	92.6
	Visual loss	38	70.4
	Facial skin infarction/ulceration	25	46.3
	Nasal congestion ± epistaxis	22	40.7
	Cavernous sinus thrombosis	22	40.7
	Cranial nerve involvement	14	25.9
	Conscious level	6	11.1
Pre-operative imaging	CT only	37	68.5
	MRI only	4	7.4
	CT and MRI	13	24.1
Surgical approach	External debridement	20	37.2
	Endoscopic	19	37
	Combined	13	24.1
	Passes away before surgery	2	3.7
Surgical serious decision	Maxillectomy	5	9.3
	Orbital exenteration	13	24.1
Systemic antifungal		50	92.6
Pathogen	Aspergillus fumigatus	12	22.2
	Mucormycosis	42	77.8
Outcome	Survivors	32	59.3
	Death	22	40.7

^*^Multiple patients had multiple symptoms

^*^Sinonasal infarction detected in all patients by endoscopy 54/54

**Table 2:** Frequency of comorbidity in COVID-19 patients with AIFRS

Comorbidity		Frequency	Percent
No comorbidity		13	24.1
Comorbidity		41	75.9
DM	Known	44	81.5
	Newly diagnosed	8	14.8
	Not diabetic	2	3.7
HTN		27	50
COPD		11	20.4
CKD		10	18.5
Haematologic malignancy		2	3.7
Hepatic		2	3.7

Clinical presentation is variable but often dramatic, with the rapid development of unilateral facial pain, tingling, numbness in the malar areas (92.6%), extension into the orbit resulting in proptosis, deterioration in vision or even visual loss (70.4%), facial skin infarction, and ulceration in 46.3%. Cerebral extension resulted in disturbed conscious level in 6 patients (11.1%) (Table 1).

Surgical debridement was done for 52/54 patients; 37.2% of patients had external debridement, 37% had endoscopic debridement, and 24.1% had combined open and endoscopic debridement, two patients passed away before surgery (Table 1). All patients who underwent surgical treatments had sinonasal tissue infarction. The aim of the surgery was to excise the necrotic tissue till healthy tissue with fresh bleeding is encountered. Beyond endoscopic debridement, maxillectomy was done in 5 patients and orbital exenteration in 13 patients (24.1%). Systemic antifungal was prescribed for 50 patients on diagnosis and was continued in the post-operative period. Mucor species was the most isolated fungal pathogens (77.8%) followed by Aspergillus fumigatus (22.2%) (Table 1). The overall mortality in the study group was 40.7% (Table 1).

**Fig. 1:**
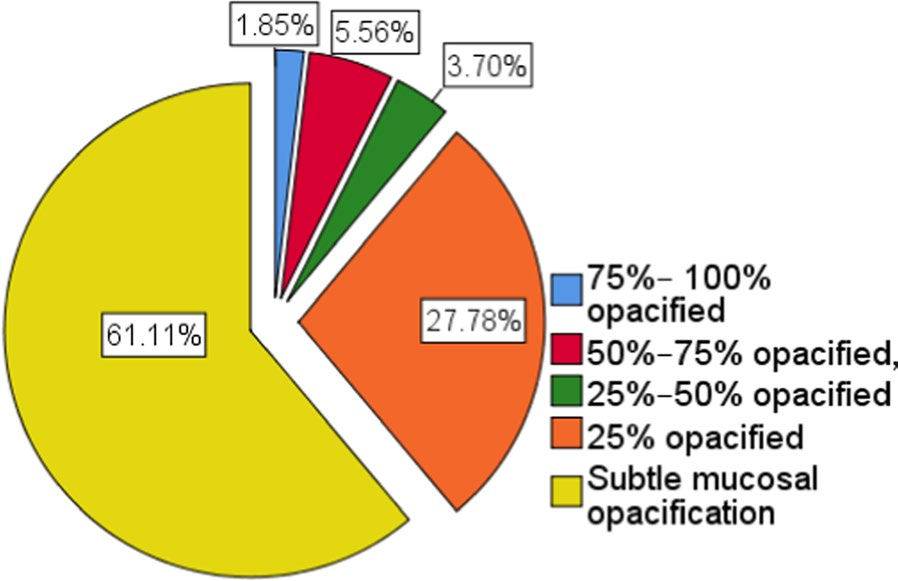
Degree of mucosal opacification of sinuses, nasal cavity, and nasopharynx

**Fig. 2:**
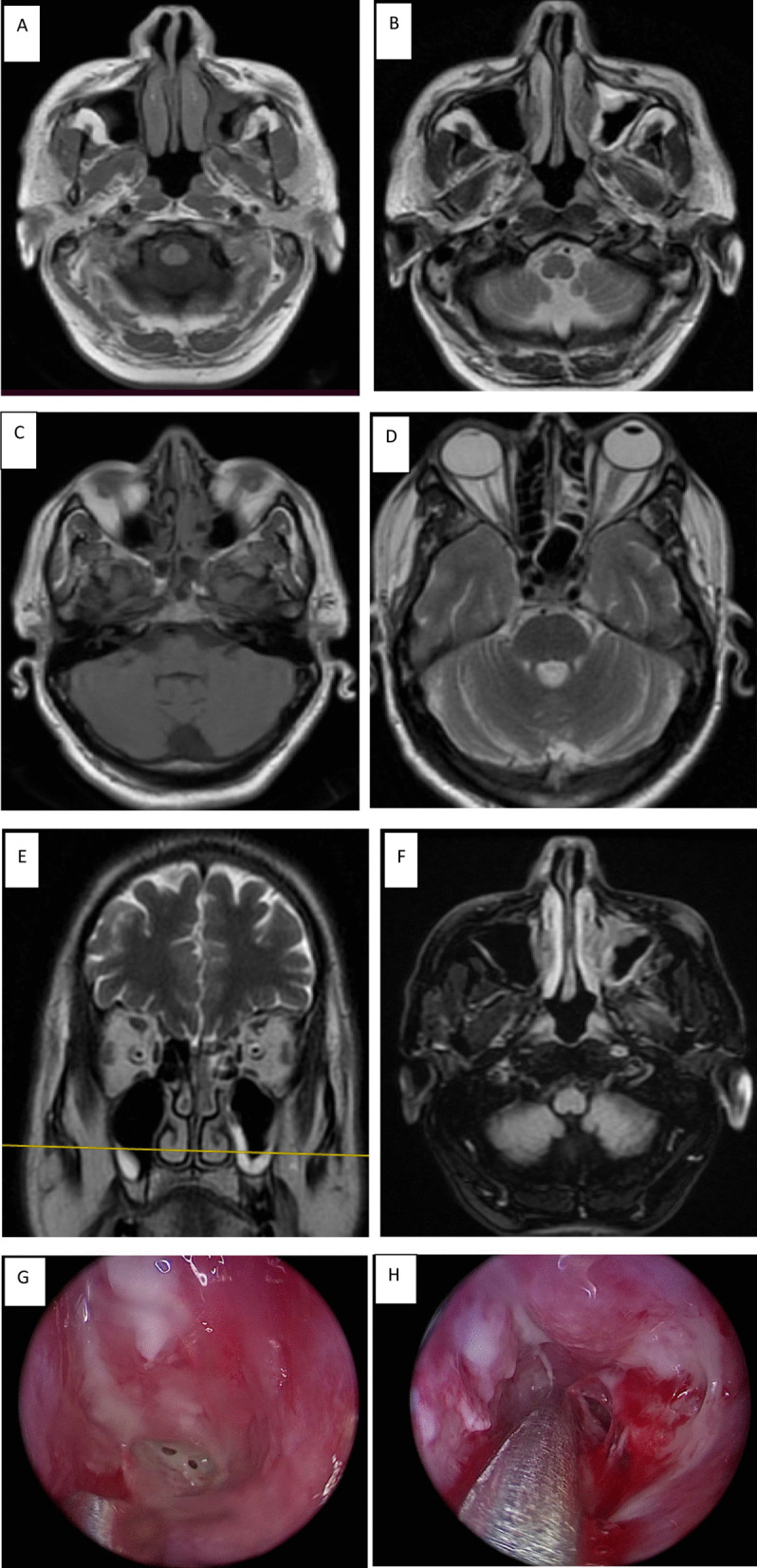
A 62-year-old diabetic female patient presented with facial tingling after 2 weeks of COVID-19 infection. **A**, **C** MRI axial T1WI **B**, **D** MRI axial T2WI, **E** coronal T2WI and **F** axial fat suppression images show subtle mucosal thickening of left maxillary sinus, left nasal cavity, and ethmoidal air cells without extension beyond the sinonasal area. Clinical examination revealed pale mucosa. Endoscopic debridement was done based on clinical suspicious and strong positive history. Fungal culture revealed definitive Mucormycosis infection. **G**, **H** 0-degree endoscopic views show pale oedematous mucosa with early necrosis of the left middle meatus

Mucosal opacification degree of the frontal, maxillary, sphenoidal, anterior ethmoidal, posterior ethmoidal, anterior nasal cavity, posterior nasal cavity and nasopharynx was reported (Fig. 1). The degree of opacity had relatively poor sensitivity and specificity as predictors for AIFS. In particular, 61% of the patients with subtle/insignificant mucosal thickening in the nasal cavity or nasopharynx, while 100% of patients showed sinonasal soft tissue infarction at endoscopy/surgery (Fig. 2). Unilateral predominance existed. Bilateral AIFR was present in one case with a strong predilection of the disease on the left side.

AIFR correlated most strongly with involvement in the pterygopalatine fossa (90.7). Surprisingly, infiltrations of pterygopalatine fossa (90.7%) preceded radiologically significant mucosal opacification of sinuses nasal cavity and nasopharynx (39.9%) (Table 3). The correlation between mucosal opacification and infiltrations was very weak; Pearson's R correlation coefficient was approximately + 0.026, and this was statistically insignificant (*p* = 0.8) (Table 4).

**Table 3:** Extrasinus soft tissue infiltration in COVID-19 patients with AIFRS enrolled in the study

Infiltration (extension beyond the sinus)	Frequency	Percent
Pterygopalatine infiltration	49	90.7
Anterior periantral fat	8	14.8
Posterior periantral fat	8	14.8
Nasolacrimal duct	4	7.4
Lacrimal sac	4	7.4
Orbital fat (medial/inferior)	16	29.6
Nasal septal mucosal ulcer	17	31.5

**Table 4:** Relation between total mucosal opacification and degree of extrasinus infiltrations

Number		Infiltration						Total
		0	2	3	4	5	7	
Total mucosal opacification	Normal	1	19	7	3	2	1	33
	25% opacified	2	9	3	0	1	0	15
	25–50% opacified	0	1	0	1	0	0	2
	50–75% opacified,	0	1	1	0	1	0	3
	75–100% opacified	0	1	0	0	0	0	1
Total		3	31	11	4	4	1	54

**Fig. 3:**
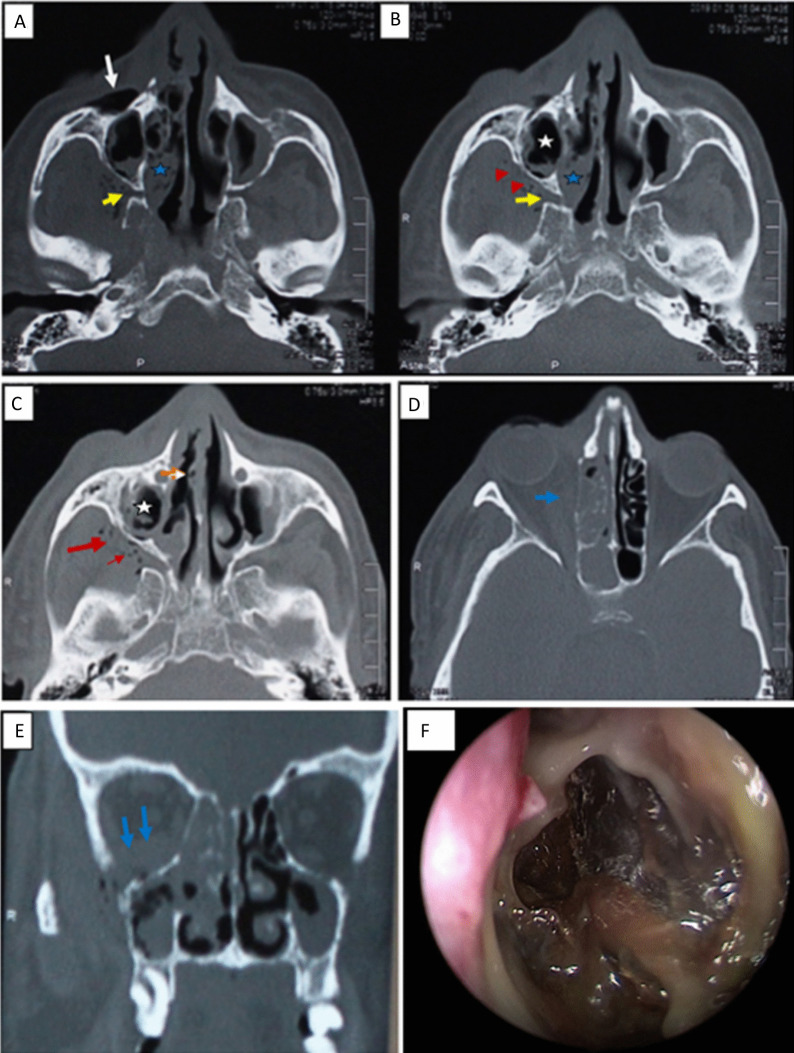
A 65-year-old male patient presented with right facial pain and swelling of 10 days duration after 15 days of COVID-19 infection with poor diabetic control. **A** Axial CT image shows mucosal thickening involving the right maxillary sinus (white asterisk) with soft tissue infiltration and gas bubbles in the right anterior periantral fat (arrow) and the posterior periantral fat (red arrowhead). **B** Axial image in the patient shows right nasal cavity mucosal thickening (blue asterisk). Soft tissue infiltration through the right sphenopalatine foramen and pterygopalatine fossa (yellow arrow) is seen, as well as involvement of the right posterior periantral fat (red arrowheads). **C** Axial CT illustrates orbital involvement of with infiltration of the right medial and inferior extraconal orbital fat (blue arrows). **D** Axial CT shows a surgically proved subtle ulceration along the left side of the nasal septum (orange arrow). **E** Coronal CT confirms orbital fat involvement. **F** endoscopic view shows marked gangrene and necrosis of right nasal cavity, nasal septum, middle and inferior turbinate. Sinus surgical exploration revealed severely involved right nasal cavity and maxillary sinus, fungal culture revealed Mucor infection, patient survived after proper diabetic control and IV amphotericin B administration

**Fig. 4:**
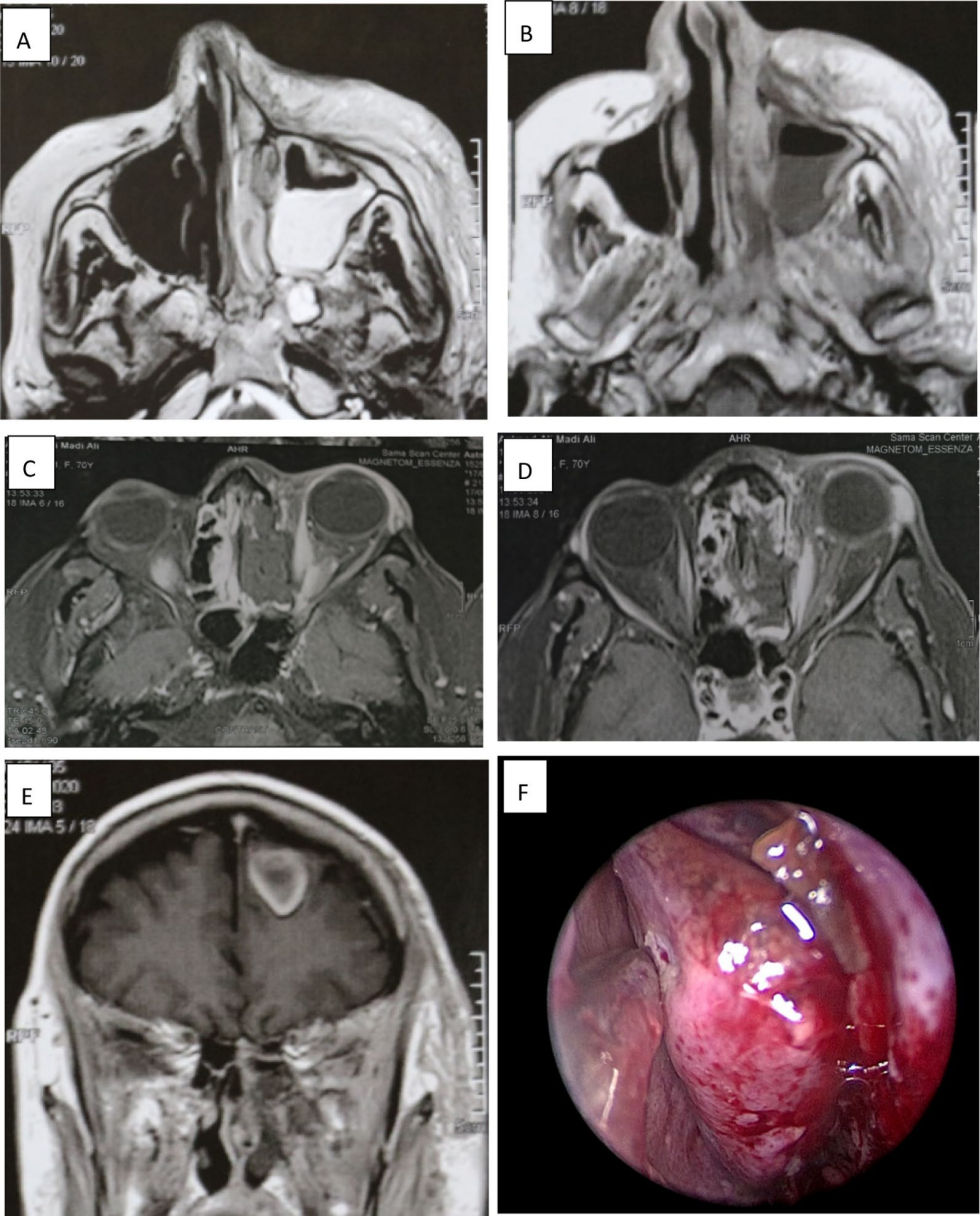
A 70-year-old female presented with left proptosis, visual loss, total ophthalmoplegia, severe headache and left facial gangrene after 4 weeks of COVID-19 infection with poor diabetic control. **A** MRI axial T2WI shows mucosal opacification of maxillary, left nasal cavity. **B** Contrast-enhanced T1WI shows enhanced mucosa with soft tissue thickening. **C**, **D** T2 with fat suppression shows mucosal thickening of the ethmoidal air cells, Infiltration medial orbital fat. **E** Contrast-enhanced T1 shows ring-enhanced brain lesion (brain abscess). **F** Endoscopic view shows necrosis and gangrene of the nasal turbinate and lateral nasal wall. Patient received treatment in the form of left maxillectomy, orbit exenteration, and abscess drainage and IV amphotericin B but patient died after 2 weeks of surgical treatment

**Fig. 5:**
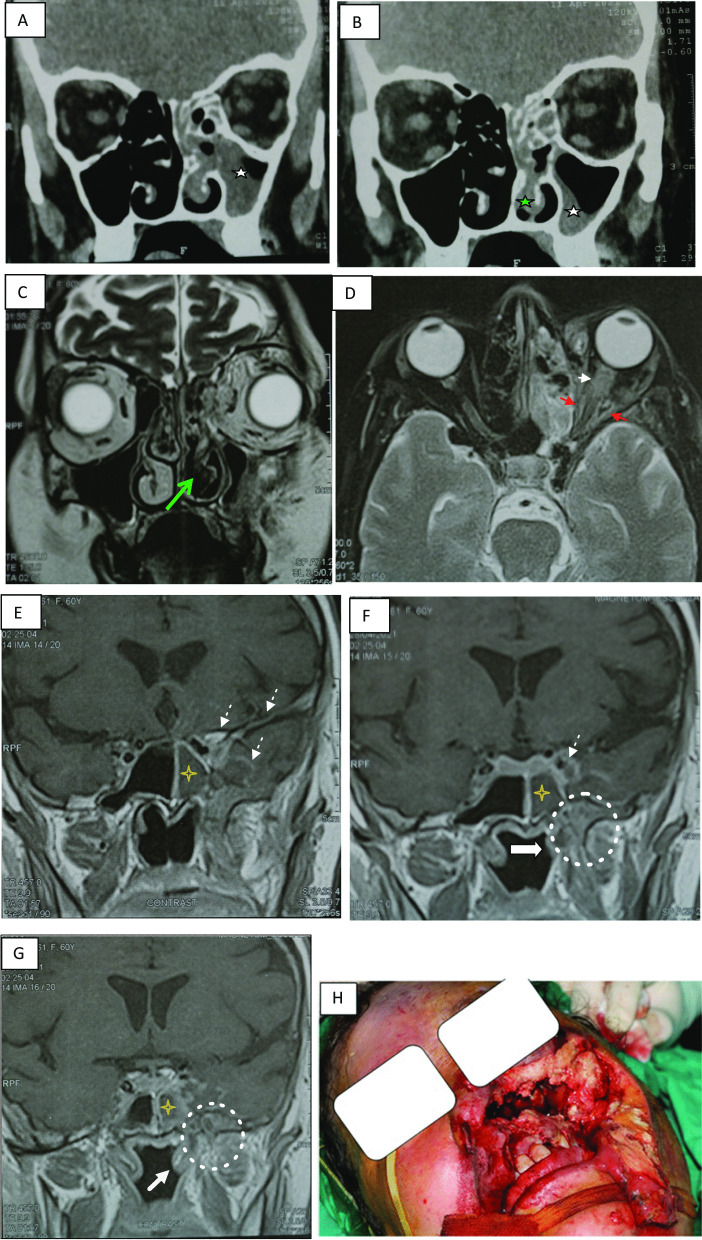
A 60-year-old female patient presented with left proptosis, visual loss, total ophthalmoplegia and left facial pain after 3 weeks of COVID-19 infection with poor diabetic control. **A**, **B** CT coronal images show mucosal thickening of the left maxillary sinus (white asterisk) and left nasal cavity (green asterisks). **C** MRI coronal T2WI shows stranding in the orbit fat, involvement of left nasal cavity (black turbinate sign). **D** MRI axial contrast-enhanced T2WI with fat suppression image shows non-enhancement and thickening of left optic nerve (white arrowhead), hypointense soft tissue intensity in left orbital apex (red arrowheads) with orbital fat stranding. **E**–**G** Coronal contrast-enhanced T1WI shows left leptomeningeal thickening and enhancement along the floor of the middle cranial fossa and abnormal enhancement in the left temporal lobe, left cavernous sinus (dashed arrows). Meckel’s cave is infiltrated with extension along the left mandibular nerve (white dashed circle) to infratemporal fossa, mucosal thickening of the left sphenoid sinus (yellow asterisks), and nasopharynx (thick arrow). **H** Operative view shows necrosis of the facial soft tissue and sinus. Patient underwent aggressive debridement in the form of left total maxillectomy and left orbit exenteration, patient survived after proper diabetic control and IV amphotericin B administration

Nasal septal ulceration was reported in 17/54 (31.5%) (Fig. 3). Orbital involvement in 29.6% (Table 4, Figs. 4, 5), intracranial involvement (epidural, subdural abscess, venous thrombosis, arterial thrombosis, cavernous sinus involvement, and intraparenchymal extension (Table 5, Fig. 5), and cranial nerves involvement were recorded in 14 patients; optic and trigeminal nerves were the most involved nerves either by direct invasion or by neuroinvasion (Table 5, Fig. 5).

**Table 5:** Extension beyond the sinus to the intracranium

Sino-cerebral extension		Frequency	Percent
Intracranial extension of infection	Subdual	18	33.3
	Epidural	18	33.3
	Brain parenchymal	22	40.7
Cavernous sinus involvement		22	40.7
Other vascular complication	Venous thrombosis	20	37
	Arterial thrombosis	1	1.9
	Both arterial and venous thrombosis	2	3.7
Cranial nerve involvement		14	25.9
Bone dehiscence/abscess		22	40.7

## Discussion

AIFRS is a severe and life-threatening condition. In this study, a systematic reporting approach is used by both the reading otolaryngologist and the reading radiologist to understand the clinical-radiological relevance of findings. Patients had a wide range of imaging characteristics. It varied from subtle mucosal thickening of sinuses up to cerebral extension. In our patients, sinonasal soft tissue necrosis was detected in 54/54 patients, inflammatory soft tissue changes extending from the sinuses into the facial subcutaneous tissue (25/54) as well as into the infratemporal and in 49/54 patients into the temporal fossa. Orbital (16/54 patients) and cerebral involvement (22/54 patients) were frequent in our study leading to neurological complications and bony destruction in 22/54 cases; cranial nerve involvement was not uncommon (14 patients).

The degree of mucosal opacification of sinuses and nasal cavity and nasopharynx had relatively poor sensitivity and specificity. Extrasinus soft tissue involvement correlated most strongly with involvement in the pterygopalatine fossa. The correlation between mucosal opacification and infiltrations was very weak. Unilateral predominance existed. Bilateral AIFR was present in one case with a strong predilection of the disease on the left side. Nasal Septal ulceration was reported in 31.5% only. We found unenhanced mucosal areas at the middle turbinate (black turbinate) as well as in the ethmoidal sinuses in 10 patients,

The CT findings presented hypodense opacification of the sinuses, unlike chronic fungal infections where the sinuses are hyperdense due to the build-up of mineral-rich fungal waste products. The cross-sectional imaging features of AIFRS associated with COVID-19 infections do not differ from those reported in the literature for AIFRS associated with other risk factors [8]. This is in concordant with the study of Middlebrooks et al. [1, 7].

The black turbinate sign described by Baumgartner et al. [9] refers to a lack of contrast enhancement of invaded mucosa due to occlusion of small vessels. The sign could also be called the black mucosa sign.

Middlebrooks et al. [7] recently proposed a simple and robust diagnostic model to serve as an easily applicable screening tool for at-risk patients with 23 variables that allow for three levels of involvement: (1) nasal cavity, paranasal sinuses, (2) rhino-orbital disease, rhino-orbito-cerebral. MRI contrast administration allows delineation of subtle areas of invasion, recognition of necrosis, and thrombosis of structures such as the cavernous sinus [1, 10, 11].

Seo et al. [12] found that (74%) of patients already had sinonasal soft tissue infarction. 100% of patients had intrasinonasal infarction and 13/17 patients also had extrasinonasal infarction and directly died of disease. Various locations of extrasinonasal infarction, including the orbit (*n* = 8/17), infratemporal fossa (*n* = 7/17), intracranial cavity (*n* = 3/17), and oral cavity and/or facial soft tissue (*n* = 4/17). Variable signal intensities were noted in the area of sinuses on T1- and T2-weighted images. Bone destruction was found on CT scans in 3/17 patients.

Ashour et al. [8] examined 8 patients and record extrasinus extensions as follows: pterygopalatine fossa (*n* = 5/8), and periantral fat (*n* = 7/8) were also noticed as well as bone dehiscence (*n* = 7/8), septal ulceration (*n* = 7/8). Bilateral disease detected in (*n* = 5/8). Orbital infiltration was unilateral in 4/8 patients. Intracranial complications were: perineural spread (*n* = 6/8), cavernous sinus involvement (*n* = 6/8), meningeal/epidural infiltration (*n* = 3/8), ICA thrombosis (*n* = 4/8), intracerebral abscess (*n* = 2/8), and a high mortality rate of 37.5%.

In the current study, we obtain the mean age of 48.06 ± 16.5 years younger than that recorded by Shintani in the USA [13]. The mean age was 58.0 ± 2.2 years, with nearly the same sex predilection of 55.9% male (55.6% male, 44.4 female).

The most common presenting symptoms of patients with AIFRS were unilateral facial pain, tingling, and numbness in the malar areas 92.6% in concordance with Turner et al. [14] who recorded facial swelling (64.5%), fever (62.9%), and nasal congestion (52.2%. Extension into the orbit results in proptosis, deterioration in vision or even visual loss (70.4%), facial skin infarction, and ulceration in 46.3%. In addition, Yin et al. [15] described that unilateral facial swelling, pain, or erythema were the most common presentations, involvement of the orbit or pterygopalatine fossa on imaging, and mucosal necrosis is seen on endoscopy.

As expected, the most commonly associated predisposing conditions were diabetes mellitus in concordance with the study of Malleshappa et al. [16] found that 78.4% patients were diabetic in concordance with the study of Wandell et al. [17] aggressive infection occurring in immunocompromised patients.

In this study 2/54 patients had haematologic malignancy on the contrary Malleshappa et al. [16] found that 17.6% had haematologic malignancies.

Mucor species (77.8%) was the most commonly isolated fungal pathogens followed by Aspergillus fumigatus (22.2%). As Raab et al. [6] pointed out, Mucorales is not the only fungi, which can infect the sinuses, Aspergillus spp. can also lead to invasive fungal sinusitis.

The aim of surgery is to excise the necrotic tissue thus maxillectomy is done in 5 patients and orbital exenteration in 13/54 patients. Systemic antifungal was described for 50 patients. This is discordant with Turner et al. [14], who described external debridement for 37.2%, endoscopic debridement in 37% and combined open and endoscopic debridement for 24.1% and Malleshappa et al. [16] who recorded partial/total maxillectomy (29.4%), orbital exenteration (7.8%) and craniotomy (2%), while Allensworth et al. [18] found that ten patients underwent maxillectomy, six with orbital exenteration. In concordance with a study done by Yin et al. [15], most patients were treated with a combination of intravenous antifungal medication and surgery. Two patients passed away before surgery.

Despite improvements in medical and surgical therapy, survival remains limited. The overall mortality in the AIFR was 40.7% in concordance with the study of Turner et al. [14] who stated an overall survival rate equalled 49.7% and Allensworth et al. [18] recorded (68%) survived which was nearly the same mentioned in the study of Malleshappa et al. [16] that gave a survival rate of 68.2% overall.

Strength of the study, up to our knowledge and the published data, is that it had a large sample and our patients were a good representative of the medical problem. Second, multidisciplinary reporting expanded the scope of data analysis.

AIFRS in COVID-19 patients is a matter of urgency; consequently, this study had some limitations. First, it was a single institutional study. Second, patient selectivity bias was possible due to rapid disease progression and poor prognosis. Third, the lack of a comparator control group limited our findings. Finally, the availability of long-term clinical outcomes was modest. The international longitudinal study is necessary for future studies.

## Conclusions

The radiological signs of AIFRS in COVID-19 patients largely vary. Inflammatory involvement outside the paranasal sinuses into facial or orbital soft tissue is the hallmark. The suspected diagnosis should be directly given to the referring physician and the medical microbiological laboratory to direct diagnostic pathways and treatment.

## Data Availability

The datasets used and/or analysed during the current study are available from the corresponding author on reasonable request.

## Abbreviations

AIFRS: Acute invasive fungal rhinosinusitis
MRI: Magnetic resonance imaging
NCCT: Non-contrast computed tomography
PNS: Paranasal sinuses
DM: Diabetes mellitus
CKD: Chronic kidney disease
HTN: Hypertension
